# Typhoid Responsibility

**Published:** 1916-01

**Authors:** G. Henri Bogart

**Affiliations:** Shelbyville, Ill.


### Typhoid Responsibility.

BY DR. G. HENRI BOGART, SHELBYVILLE, ILL.

The claim of the brotherhood of man as exemplified in the story of the good Samaritan has just had two unusual advances in the higher courts. Three children contracted typhoid fever because the public water supply of the city of Mt. Holly, supplied by a private corporation, had become contaminated.

The father brought suit for damages and the verdict for $750 damages was appealed to the Supreme Court of New Jersey. The verdict was sustained, the court ruling:

"It must be borne in mind that the defendant water supply company was in business for profit. The plaintiff had paid in advance for water supply. Hence, it became the duty of the defendant company to give to the plaintiff water fit for domestic supply, including fitness for drinking. Water is a necessity of life, and one who undertakes to trade in it and to supply customers stands in no different position to those with whom he deals than does a dealer in foodstuffs.

"Actual knowledge of the unwholesomeness of the water was not an essential element to be proven in order to establish the defendant's liability.

"The defendant, in the exercise of reasonable care, might have discovered the unwholesomeness and dangerous condition of the water."

Directly upon the heels of this decision comes another from Wisconsin. The drinking water supplied to the workmen employed in a lumber company's plant was infected with typhoid germs and the men contracted the disease. Suit was brought for recompense, under the workmen's compensation law, and was carried to the Supreme Court, the ruling being that death or inability to work resulted from an accident for which the company is responsible.

The far-reaching results of these two decisions are more startling than the decisions themselves. They bridge the chasm of no responsibility more than half-way to a point where the State shall be held directly responsible for any death or sickness from any preventable disease.

---

All narcotics have an affinity for fats, and as fats predominate in the brain cells the brain is first affected by narcotics. But why this elective affinity?—*J. W. Williams, Charlotte Medical Journal.*

